# Pilot study of DNA methylation, molecular aging markers and measures of health and well-being in aging

**DOI:** 10.1038/s41398-019-0446-1

**Published:** 2019-03-18

**Authors:** Chirag M. Vyas, Aditi Hazra, Shun-Chiao Chang, Weiliang Qiu, Charles F. Reynolds, David Mischoulon, Grace Chang, JoAnn E. Manson, Immaculata De Vivo, Olivia I. Okereke

**Affiliations:** 1 Department of Psychiatry, Massachusetts General Hospital and Harvard Medical School, Boston, MA USA; 2 Channing Division of Network Medicine, Department of Medicine, Brigham and Women’s Hospital and Harvard Medical School, Boston, MA USA; 3 Division of Preventive Medicine, Department of Medicine, Brigham and Women’s Hospital and Harvard Medical School, Boston, MA USA; 4 Department of Psychiatry, UPMC and University of Pittsburgh School of Medicine, Pittsburgh, PA USA; 5 Department of Psychiatry, VA Boston Healthcare System, Brockton, MA, USA and Harvard Medical School, Boston, MA USA; 6 Department of Epidemiology, Harvard T.H. Chan School of Public Health, Boston, MA USA

## Abstract

Relations of DNA methylation markers to other biological aging markers and to psychosocial, behavioral, and health measures remain unclear. The sample included 23 participants (*n* = 11 cases with psychiatric diagnoses and *n* = 12 controls without current or lifetime psychiatric disorder), balanced by age and sex. Genomic DNA was extracted from blood samples; the following were performed: genome-wide DNA methylation assay using Illumina 850k methylationEPIC; PCR assays for relative telomere length (RTL) and mitochondrial DNA copy number (mtCN). Exposures were: case status; depression and anxiety symptoms; psychosocial support; subjective and objective cognition. Outcomes were: DNA methylation age (DNAm age); RTL; mtCN; extrinsic and intrinsic epigenetic age acceleration (EEAA and IEAA). Stronger correlation with chronological age was observed for DNAm age (*ρ* = 0.86; *p* < 0.0001) compared to RTL (*ρ* = −0.53; *p* < 0.01); mtCN was not correlated with age. DNAm age was more strongly correlated with behavioral and health variables than RTL or mtCN; e.g., correlations with DNAm age: body mass index (*ρ* = 0.36; *p* = 0.10); smoking pack-years (*ρ* = 0.37; *p* = 0.08); physical activity (*ρ* = −0.56; *p* = 0.01); alcohol intake (*ρ* = 0.56; *p* = 0.01). DNAm age was inversely correlated with psychosocial support (*ρ* = −0.42; *p* = 0.048) and Modified Mini-Mental State score (*ρ* = −0.44; *p* = 0.01). Anxiety, psychosocial support, and objective cognition were significantly related to accelerated aging; depression and subjective cognition were not. In conclusion, DNAm age correlated more strongly with chronological age and key psychosocial, behavioral, and health variables than RTL or mtCN. Signals for associations with epigenetic aging were observed for psychosocial and neurobehavioral variables.

## Introduction

Knowledge gaps exist regarding the biological mechanisms that are involved in trajectories of health and aging. DNA methylation—the addition of a methyl group to the CpG dinucleotide—is an epigenetic mechanism involved in switching genes on and off (gene expression), with the potential to offer critical insights regarding mechanisms of stress, behavior, and aging. DNA methylation markers may also enrich the current complement of biological aging markers, such as telomere length and mitochondrial DNA copy number (mtCN)—both of which have previously been associated with aging and/or diseases of aging^1,2^. Epigenetic changes may reflect influences of psychosocial, behavioral, and health risk factors relevant to aging and have also been implicated in psychiatric disorders^3^. Moreover, epigenetic aging—or the epigenetic “clock”—is a novel and powerful construct that strongly relates to aging across multiple tissues and organ systems in humans^4,5^. Variation in epigenetic aging has been associated with age-related diseases and overall mortality^4–8^. Recently, Horvath and co-workers identified and validated a group of 353 CpG markers for evaluating the epigenetic clock across 51 human healthy tissues and cell types and developed an algorithm to estimate chronological age in healthy persons^4–6,9^.

For numerous reasons—e.g., psychological stress, medical illness, lifestyle factors—a mismatch can occur between DNA methylation age (DNAm age) and a person’s chronological age^5,10,11^. Thus, the difference between methylation-predicted age and chronological age serves as an index of disproportionate or accelerated biological aging^5^. Indeed, DNAm age of white blood cells in peripheral blood samples has recently been associated with psychiatric illnesses, such as bipolar and major depressive disorder (MDD)^12,13^. Similarly, clinical-level psychiatric symptoms or disorders have been significantly associated other biological aging markers, such as telomere length^14–17^ and mtCN^12^. Nevertheless, this remains an emerging literature, and there have been few studies that evaluated the relationship of these aging markers with respect to both each other as well as a broad range of psychosocial, lifestyle and health variables—including modifiable ones.

Therefore, this pilot study leverages the high-quality data of well-characterized participants in an ongoing study of late-life depression to measure three aging biomarkers (DNAm age, telomere length, and mtCN). We evaluated the associations of these markers with each other as well as the relationship between epigenetic and molecular markers of aging and key psychosocial, behavioral and health measures.

## Methods

### Source of participants

Participants were from VITAL-DEP (VITamin D and OmegA-3 TriaL-Depression Endpoint Prevention, NCT01696435), a late-life depression prevention ancillary study^18,19^ to the VITAL (NCT01169259) trial^20,21^. VITAL consists of 25,871 men and women, aged 50+ and 55+ years (mean = 67 years), respectively, in a 2 × 2 factorial randomized trial of heart disease and cancer prevention using vitamin D and/or fish oil; cohort details are provided elsewhere^20^. VITAL also incorporates a Clinical Translational Science Center (CTSC) sub-cohort of *n* = 1054 men and women, all of whom are participants in the full trial, who presented for baseline and 2-year follow-up visits. Among these CTSC participants, VITAL-DEP established a set of *n* = 1041 adults who completed comprehensive neuropsychiatric assessments at baseline and 2-year follow-up, as detailed elsewhere^19^. Participants for the current study are members of the VITAL-DEP CTSC component.

### Sample selection

We selected 23 participants (*n* = 11 cases and *n* = 12 controls). Cases were diagnosed with DSM (Diagnostic and Statistical Manual)-IV-criteria psychiatric disorders^22^, as determined by the Mini-International Neuropsychiatric Interview (MINI)^23^ during the VITAL-DEP CTSC component protocol. Cases included those with: bipolar disorder (*n* = 2), unipolar major depression (*n* = 2), phobic anxiety (panic disorder, social anxiety disorder, agoraphobia; *n* = 4), post-traumatic stress disorder (*n* = 1), co-morbid unipolar depression and panic disorder (*n* = 1), and co-morbid bipolar, obsessive-compulsive disorder and panic disorder (*n* = 1). Controls were healthy persons with no current or lifetime history of DSM-IV criteria psychiatric disorder. Cases and controls were balanced by sex and age (i.e., distributions of 10-year age groups from 50–90 years) and were selected form a larger subset of participants who had both existing telomere and mtCN data (see below). These selected 23 CTSC participants also had complete blood counts (CBCs) and at least 1 μg of extracted genomic DNA available. Participants with extreme characteristics that could bias the methylation results (e.g., heavy/binge alcohol use) were excluded from this selection.

### Psychosocial, behavioral, and health measures

As above, DSM diagnoses were obtained during CTSC-based interviews using the MINI. Self-report instruments were completed by all participants to capture behavioral symptoms, risk factors or risk modifiers in late-life depression. These were: Patient Health Questionnaire-9 (PHQ-9);^24^ Generalized Anxiety Disorder-7 (GAD-7);^25^ the Duke Social Support Index (DSSI), a validated scale that incorporates both frequency of social supports and contacts as well as satisfaction with social support and level of contact;^26,27^ the STIDA (Structured Telephone Interview for Dementia Assessment)^28^ self-report questions, which assess subjective cognitive concerns. Finally, objective cognitive performance was assessed using the 3MS (Modified Mini-Mental State) and other tests of individual domains (memory, attention, semantic fluency, and executive function)^29,30^. Because of its particular relevance to aging, we derived delayed memory *z*-scores by averaging delayed recall trials of the East Boston Memory Test paragraph recall and a 10-word recall^30,31^.

### Biomarker assays

Baseline blood samples were collected in the CTSC prior to randomization into VITAL. Genomic DNA was extracted from peripheral blood leukocytes (buffy-coat cryotubes had been stored in the vapor phase of liquid nitrogen freezers at ≤−130 °C for later use) using the QIAamp® DNA Blood Mini Kit (Qiagen Inc., Valencia, CA), and PicoGreen DNA quantitation was performed using a Molecular Devices 96-well spectrophotometer. We performed DNA methylation, telomere length, and mtCN assays using leukocyte DNA.

### DNA methylation assay

DNA methylation assays on participant samples and quality controls (QCs) were conducted using the Illumina Infinium MethylationEPIC BeadArray technology (Methyl850K chip) that allows genome-wide DNA methylation analysis of 866,836 CpG sites^32^. Bisulfite conversion, or bisulfite treatment, whole genome amplification (WGA), hybridization and single base extension were carried out to get labeled nucleotide. Dual color (Cy3 and Cy5) staining allows the nucleotide to be detected by the iSCAN reader and is converted to genotype during analysis using pre-processing software programs (e.g., *minfi*) available in R programming language. Raw methylation data was normalized using Noob-normalization with the *minfi* package in R^33,34^.

### Relative telomere length (RTL) assay

RTL assays were conducted as part of a larger study that included 800 VITAL-DEP participants who were selected to balance age (evenly distributed across 10-year age groups between ages 50–90 years), race/ethnicity and sex; these assay results were leveraged for the current study. Briefly, we measured RTL using quantitative real-time PCR. Average RTL was calculated as the exponentiated ratio of Telomere repeat copy number to Single gene (36B4) copy number (T/S) corrected for a reference sample, as described elsewhere^35^. Laboratory technicians were masked to participant characteristics and assayed each sample in triplicate. QC samples were interspersed on each plate to assess variability. Although this assay provides a relative measurement of telomere length, T/S ratios are known to correlate well with absolute telomere lengths determined by Southern blot (*r* = 0.82, *p* < 0.0001)^35^.

### Mitchondrial DNA copy number assay

As with the RTL assays, MtCN assays were also conducted as part of a larger study that included 450 VITAL-DEP participants who were similarly selected to balance age, race/ethnicity and sex, and overlapped with the above-noted participants in the RTL study; results were leveraged for this study. Briefly, this is a high-throughput quantitative PCR (qPCR)-based assay involves a multiplex ND2 (single-copy mitochondrial gene) and AluYb8 (nuclear repeat element) PCR reaction mixture. Triplicate reactions of multiplex reactions were performed on each sample on different plates. Samples were run in a single batch, to reduce batch variation effects. The qPCR-based assay determined the mitochondrial ND2 gene copy number to genomic single-copy gene copy number (N/S) ratio, a value proportional to the average number of mtDNA copy number. Detailed procedures for the mtCN assay are described elsewhere^36,37^.

### Sample quality control

To assess QC of the DNA methylation assays we included two blinded samples, with random placement on the plate, in duplicate for testing of QC replicates, as well as one in-lab genotyping control. The sample quality curve showed that: samples passed the QC threshold; genotyping was successful; biological sex (X, Y chromosome) was correctly identified. The expected bi-modal distribution (reflecting two populations of predominantly methylated vs. unmethylated sites) was seen. CpG methylation values in the *n* = 2 pairs of QC replicates were highly correlated (*r* = 0.98). Finally, we found high correlations between estimated cell counts using our methylation assay data and the known CBCs with differential measured in the hospital lab when participants arrived in the CTSC: Spearman rho *(ρ)* values ranged from 0.60 to 0.75 (*p*-values < 0.01) for lymphocytes, granulocytes, and monocytes. Similarly, blinded replicates were included among samples for the RTL and mtCN assays and were randomly distributed across the plates. The average coefficient of variation (CV) for was 6.6% for T/S ratio; for the mtCN assay the average CV was 7.0%.

### DNA methylation analysis and bioinformatics approach

We used the *minfi* Bioconductor package in R for processing functions (performing background correction using negative control probe signal intensities, as well as normalization and correction of dye imbalance) and analyzing the Illumina 850k methylationEPIC data^34^. To assess genome wide differences by case status in methylation at the CpG level, we used the ‘DMPFinder’ function. We computed DNAm age, epigenetic age acceleration (i.e., from regression of DNAm age on chronological age, or AgeAccel), extrinsic epigenetic age acceleration (EEAA), and intrinsic epigenetic age acceleration (IEAA) using methods of Horvath and Hannum (https://dnamage.genetics.ucla.edu/). The Horvath age estimation algorithm predicts DNAm age based on the methylation levels of 353 CpGs, as previously described; to improve the predictive accuracy, the online tool normalizes the methylation data^5^. AgeAccel is defined as the residual that results from regressing DNAm age on chronological age^8,11^. EEAA is correlated with immune system aging; positive values denote that epigenetic age is higher than that expected based on chronological age—i.e., accelerated epigenetic aging^11^. The IEAA is the residual resulting from a multivariate model that regresses calculated DNAm age on chronological age + cell counts^5,9,11,38^.

### Statistical analysis

Summary statistics are provided as median with IQR for non-normally distributed continuous variables and as percentage for categorical variables, stratified by case and control status. Distributions of the continuous variables were assessed by the Shapiro-Wilk test and histogram visualization. Wilcoxon *t*-test was used for non-normally distributed continuous variables. Considering the small sample size, Fisher’s exact test was used to compare all categorical variables by case/control status. We computed Spearman rank correlations across all three aging markers. Additionally, we evaluated correlations between aging markers and continuous demographic and lifestyle variables. For multiple testing hypothesis, we analyzed all correlations at false discovery rate (FDR) <0.05. We examined the associations of key exposures with the DNAm age, AgeAccel, EEAA, and IEAA by Horvath and Hannum methods^4,5^. We also examined the correlations between the aging markers (i.e., DNAm age, RTL, mtCN, AgeAccel, EEAA, and IEAA) with the psychosocial and neurobehavioral outcomes of PHQ-9, GAD-7, DSSI score, 3MS score, delayed recall *z*-score and STIDA score.

Although the primary focus of the study was to relate epigenetic and molecular markers of aging to psychosocial, behavioral, and health measures, we conducted an exploratory analysis to assess genome wide differences in DNA methylation by psychiatric case vs. control status. Annotation was performed using available tools (DAVID, GO)^39,40^; parallel boxplots were generated after identifying top genes associated with differentially methylated CpGs in cases vs. controls.

All statistical analyses were performed with SAS version 9.3 (SAS, Cary, NC). Statistical significance was defined as a two-tailed *p*-value < 0.05 for all analyses. The study was approved by the Institutional Review Board at the Brigham and Women’s Hospital.

## Results

Table 1 shows key exposures such as demographic, lifestyle, behavioral, clinical, aging markers, and psychosocial and neurobehavioral variables by psychiatric case and control status. In demographic characteristics: age, sex, race, and education were similar between the two groups; compared to control status, case status was associated with lower income (i.e., <$50,000/year vs. above) (*p* = 0.02). Regarding lifestyle modifiers, current smoking was more prevalent among those with vs. without psychiatric history (90.9% vs. 41.7%; *p* = 0.03). Other lifestyle characteristics, such as alcohol use, body mass index (BMI), history of diabetes and hypertension, and physical activity, were similar by case/control status. Comparing participants with vs. without psychiatric case status: median PHQ-9, GAD-7, 3MS, and STIDA (subject cognitive concern) scores were non-significantly higher; DSSI (self-rated psychosocial support) and delayed recall scores were non-significantly lower. Distributions of white blood cell type counts were similar for both groups. There were no significant differences in DNAm age by psychiatric case/control status [median (interquartile range, IQR) was 59.7 years (56.4–66.2) for cases vs. 59.4 years (55.7–63.1) for controls]. Similarly, there were no significant differences by case/control status for RTL or mtCN; comparing cases vs. controls, median (IQR) RTL was 0.55 (0.49–0.60) vs. 0.56 (0.51–0.60), and median (IQR) mtCN was 0.52 (0.31–0.82) vs. 0.54 (0.35–0.63).

**Table 1:** Baseline characteristics of psychiatric disorder case and control participants

Baseline characteristics	Case (*n* = 11)	Control (*n* = 12)	*p* Value
Demographic factors			
Age, median (IQR)	67.1 (60.0–74.6)	66.3 (56.4–72.5)	0.56
Sex, *n* (%)			0.68
Female	7 (63.6)	6 (50.0)	
Male	4 (36.4)	6 (50.0)	
Race/ethnicity, *n* (%)			0.64
African-American	2 (18.2)	4 (33.3)	
White	9 (81.8)	8 (66.7)	
Education, *n* (%)			
College level or less	7 (63.6)	8 (66.7)	0.99
Post-college	4 (36.4)	4 (33.3)	
Income, *n* (%)^a^			0.02
Lower income, <$50,000/year	7 (87.5)	2 (22.2)	
Higher income, ≥$50000/year	1 (12.5)	7 (77.8)	
Lifestyle and health factors			
Smoking, *n* (%)			0.03
Never or past smoking	1 (9.1)	7 (58.3)	
Current smoking	10 (90.9)	5 (41.7)	
Alcohol, *n* (%)			0.64
Daily use	2 (18.2)	4 (33.3)	
All other frequencies of intake	9 (81.8)	8 (66.7)	
Obesity, *n* (%)^a^			0.17
BMI < 30 kg/m^2^	5 (50.0)	10 (83.3)	
BMI ≥ 30 kg/m^2^	5 (50.0)	2 (16.7)	
Diabetes, *n* (%)			0.32
Yes	3 (27.3)	1 (8.3)	
No	8 (72.7)	11 (91.7)	
Hypertension, *n* (%)			0.37
Yes	9 (81.8)	7 (58.3)	
No	2 (18.2)	5 (41.7)	
Total exercise (frequency per week), median (IQR)	2.5 (1.0–6.5)	5.5 (2.8–8.5)	0.23
Physical activity (total met-HR), median (IQR)	10.0 (3.0–21.0)	25.3 (14.7–40.0)	0.1
Cell counts			
% Monocytes, median (IQR)	4.80 (3.63–6.63)	5.36 (3.17–7.35)	0.69
% Granulocytes, median (IQR)	71.04 (61.46–77.61)	64.62 (53.06–73.22)	0.2
% Lymphocytes, median (IQR)	32.23 (23.87–39.26)	36.67 (27.69–46.20)	0.35
Molecular aging markers			
DNA Methylation age, median (IQR)	59.72 (56.43–66.16)	59.35 (55.72–63.11)	0.65
Telomere length, median (IQR)	0.55 (0.49–0.60)	0.56 (0.51–0.60)	0.61
Mitochondrial copy number, median (IQR)	0.52 (0.31–0.82)	0.54 (0.35–0.63)	0.98
Psychosocial and neurobehavioral variables			
PHQ-9, median (IQR)	3.0 (0.0–7.0)	1.0 (0.0–2.5)	0.17
GAD-7, median (IQR)	1.0 (0.0–5.0)	0.5 (0.0–2.0)	0.52
DSSI, median (IQR)	20.0 (15.0–27.0)	26.0 (22.0–28.0)	0.16
STIDA, median (IQR)	1.0 (0.0–3.0)	0.0 (0.0–1.0)	0.39
3MS, median (IQR)	95.0 (90.0–98.0)	94.0 (93.0–98.0)	0.73
Delayed recall *z*-score, median (IQR)^b^	−0.3 (−0.6–0.2)	−0.1 (−0.9–0.2)	0.67

*IQR* interquartile range, *BM* body mass index, *PHQ* Patient Health Questionnaire, *GAD* Generalized Anxiety Disorder, *DSSI* Duke Social Support Index, *STIDA* Structured Telephone Interview for Dementia Assessment self-report question score, 3MS Modified Mini-Mental State

^a^Values shown for participants with non-missing values for income and BMI

^b^Delayed recall z-score is calculated based on averaging delayed verbal memory recall trials of the East Boston Memory Test paragraph and a 10-word list

**Fig. 1:**
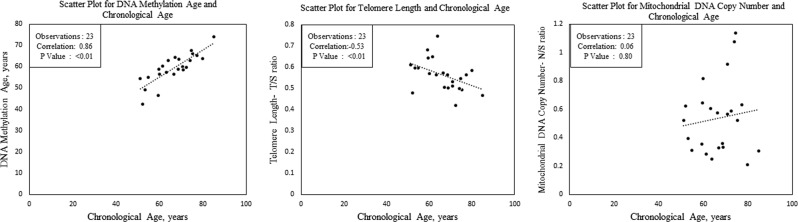
Correlation of aging markers and chronological age. Scatterplots are shown of the Spearman rank correlations between molecular aging markers and chronological age. DNA methylation age (in years) is calculated using the Horvath 353-CpG calculator

**Fig. 2:**
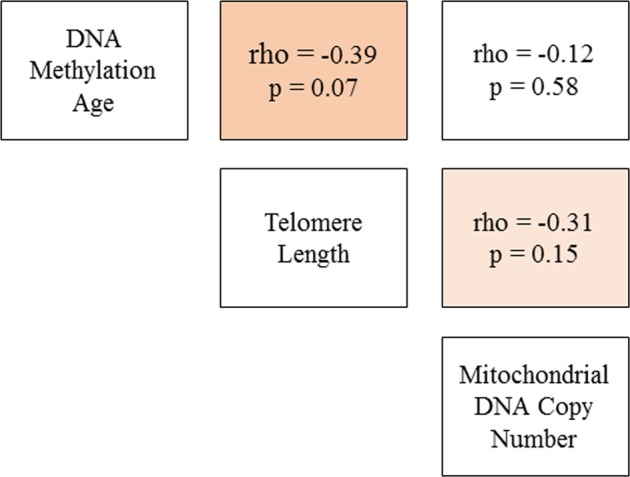
Correlations of DNA methylation age with telomere length and mitochondrial DNA copy number. Matrix of Spearman rank correlation coefficients and p-values for the three molecular aging markers. Stronger estimated correlation is illustrated by darker color

Figure 1 shows the scatterplots originating from Spearman rank correlations between molecular aging markers and chronological age. DNAm age was most highly correlated with chronological age (rho (*ρ*) = 0.86, *p* < 0.001), followed by telomere length (*ρ* = 0.53, *p* < 0.01), but there was no correlation between mtCN and chronological age. We also found a suggestion of a correlation between DNAm age and RTL (*ρ* = −0.39, *p* = 0.07; Fig. 2), but no evidence of correlation between DNAm age and mtCN.

**Fig. 3:**
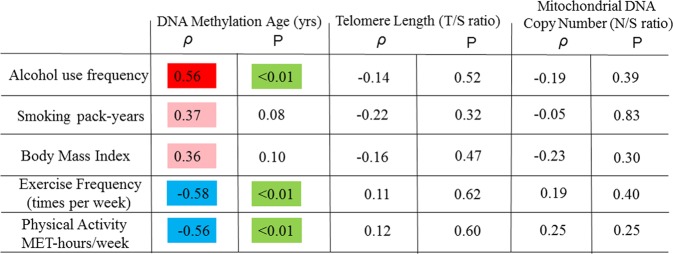
Correlation of aging markers and lifestyle characteristics. Spearman rank correlation coefficients (rho, *ρ*) and p-values for lifestyle/behavior variables and aging markers are shown. Stronger estimated correlation is illustrated by darker color. Positive correlations are depicted in red, negative correlations are in blue and p-values are in green. Multiple testing hypothesis at False Discovery Rate < 0.05 was used

Correlations between key exposures of lifestyle factors and aging markers are shown in Fig. 3. DNAm age had a modest positive correlation with alcohol use (*ρ* = 0.56; *p* < 0.01) and was negatively correlated with exercise frequency (occasions/week) (*ρ* = −0.58; *p* < 0.01) and total metabolic equivalent (MET)-hours per week (*ρ* = −0.56; *p* < 0.01). All correlations were remained significant at FDR < 0.05. We observed estimates in the direction of positive correlations of smoking pack-years and BMI with DNAm age. There were no significant associations of any of the above-noted lifestyle or health factors with RTL or mtCN in this sample of 23 participants.

Additionally, we calculated associations between baseline categorical characteristics and molecular aging markers. We did not find any significant differences of DNAm age, AgeAccel, EEAA or IEAA comparing case vs. control status. Persons with post-baccalaureate vs. lower levels of education had DNAm age nearly 9 years lower on average (*p* = 0.01); similar results were found for extrinsic and IEAA. Individuals with past or current smoking history, daily alcohol consumption (daily vs. all other frequencies of intake), and lower physical activity (<7 times vs. ≥7 times per week) had DNAm age nearly 6–8 years higher on average (all *p*-values < 0.01). Differences in DNAm age were not statistically significant for diabetes, hypertension, and obesity (BMI < 30 kg/m^2^ vs. ≥30 kg/m^2^), but estimates were in the direction of 4–5 years higher DNAm age among those with vs. without these health conditions (Data not shown in tables, but available upon request).

**Fig. 4:**
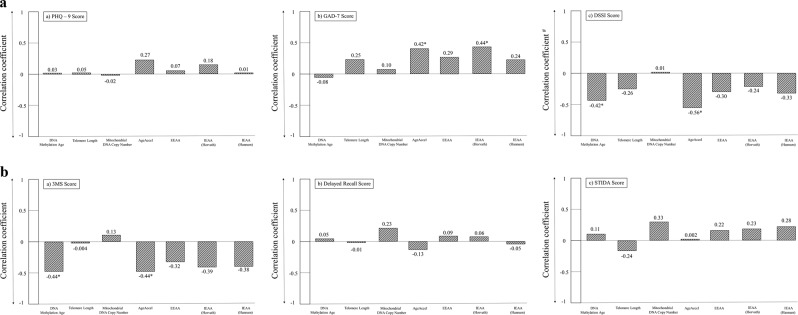
Correlations of psychological, social and cognitive measures with molecular aging markers. **a** Correlations of psychiatric symptom and psychosocial measures with molecular aging markers. **b** Correlations of objective and subjective cognitive score measures with molecular aging markers

Figure 4 shows the correlations between the psychosocial, behavioral, and cognitive variables and molecular aging markers. DNAm age was inversely correlated with self-rated psychosocial support (*r* = −0.42; *p* = 0.048) and objective 3MS cognitive score (*ρ* = −0.44; *p* = 0.01). Accelerated aging (AgeAccel) was positively correlated with anxiety (*ρ* = 0.42; *p* = 0.04) and inversely correlated with psychosocial support (*r* = −0.56; *p* = 0.01) and objective 3MS cognitive score (*ρ* = −0.44; *p* = 0.03). Depression, delayed recall, and subjective cognitive score were not significantly correlated with DNAm age or accelerated aging.

Finally, we performed a preliminary genome-wide methylation analysis of differences by case/control status in individual CpG probes and regions. Given the small sample size, this was exploratory in nature. We did not find genome-wide significant loci with FDR correction for multiple testing (adjusted *p*-value > 0.05); however, there were 76 CpGs that differed by case/control status at the *p* < 10^−4^ level, with many at *p* < 10^−5^ or *p* < 10^−6^. Using DAVID and GO functional annotation tools, we investigated these nominal top ‘hits’ (see Supplementary table). While only nominally significant at *p* < 0.05, there was preliminary indication of differential methylation of genes involved in GTPase activity (GO ID:0090630) and cell-cell adhesion (GO ID:0098609) (See Supplementary material).

## Discussion

In this pilot study, we observed that chronological age was strongly correlated with DNAm age, and moderately correlated with telomere length, but not correlated with mtCN. DNAm age was also significantly associated with demographic and lifestyle factors, such as education, exercise, alcohol intake, and smoking. Furthermore, we observed strong signals for associations of epigenetic aging with psychosocial and neurobehavioral variables. Specifically, DNAm age and accelerated epigenetic aging were inversely correlated with psychosocial support and objective cognitive performance, while anxiety symptom severity was positively correlated with accelerated epigenetic aging.

The association between DNAm age and chronological age has been reported previously^4,5^ and our current findings, even in a sample of modest size, are consistent with these prior reports. However, few prior studies had evaluated the relationship of these aging markers with each other. The estimate for the correlation between DNAm age and telomere length observed in our sample was slightly stronger than has been reported elsewhere^10,41,42^; however, DNAm age was calculated in this study using the 353-CpG probe method of Horvath^5^ rather than the 71-probe calculator of Hannum et al.^4^. In a much larger cross-sectional study, Quach et al.^11^ reported significant associations of epigenetic age acceleration with education, income as well as dietary and lifestyle factors. Although our sample size was smaller in scale than that of this prior work, we were similarly able to identify significant differences in epigenetic age by education and lifestyle factors (smoking, alcohol intake, and physical activity).

Regarding psychiatric case status, we did not find any evidence of differences in epigenetic aging among the 11 cases and 12 control participants in our study. Similarly, McKinney et al.^43^ reported that there were no differences in accelerated epigenetic aging, measured by DNA methylation, between 22 case participants with schizophrenia and 22 control participants. Nevertheless, a more recent paper by Han et al.^13^ identified significant differences in accelerated epigenetic aging, measured by DNA methylation in blood, between 811 participants with major depression vs. 319 control participants. Such findings suggest that larger sample sizes may be required to achieve adequate statistical power to identify significant differences in DNAm age metrics by psychiatric case/control status.

To our knowledge, this is the first study to suggest links between self-reported psychosocial support levels (summarizing number, frequency as well as subjective rating of closeness of contacts) and DNAm age or accelerated epigenetic aging. These findings can be placed in the context of previous studies that have reported dynamic changes in DNA methylation of stress-associated genes after acute psychosocial stress exposure^44^, influences of chronic lifetime stressors on DNAm age^45^, and the role of social adversity on epigenetic aging markers^46^.

Strengths of the study are noted. First, the project involved novel technology: Illumina Methylation EPIC 850K chip is the latest generation of genome-wide DNA methylation assay and expanded upon the widely used 450K microarray to extend coverage. Second, the study yielded novel findings regarding the significant associations of higher psychosocial support and better objective cognitive function with lower mean epigenetic age and lower accelerated aging. Finally, we able to explore potential differences in gene expression among our participants, by capturing variations in methylation levels at specific CpGs. While exploratory in nature, the functional annotation findings were consistent with the key mechanisms invoked in affective disorders: i.e., GTPase activity is fundamental in neurotransmission; most neurotransmitter receptors in the brain (e.g., serotonergic, noradrenergic, and dopaminergic) are G-protein-linked^47^. Similarly, cell–cell adhesion has relevance to synaptic transmission^48^ and to inflammatory and immune processes^49^ hypothesized in mood disorders^50–54^. Han et al.^13^ also identified similar functional annotated pathways seen in our current study.

We acknowledge limitations to this study. Although we identified compelling signals for possible associations between epigenetic markers and the psychosocial, behavioral, and health variables, the sample size was relatively small, and caution is needed regarding potential for chance findings. In addition, although the sample case/control selections were balanced by age and sex, and were drawn from the CTSC sub-cohort to reflect a broad distribution of ages, the selection has the potential to bias results; thus, replicating these approaches in a larger sample or in a separate cohort would advance knowledge regarding the reported associations in this study. There was also heterogeneity in psychiatric diagnoses of cases; thus, future work is needed to address whether there are differences in DNA methylation and epigenetic aging by specific disorders or groups of disorders. Heterogeneity of cell counts was also present, and we thus adjusted for cell count distributions in our analyses. Finally, the study was cross-sectional and had low racial/ethnic diversity; longitudinal studies with larger samples of diverse participants are necessary to confirm and clarify the nature of associations (e.g., causal, mechanistic) and to examine possible differences in associations by sex, race or ethnicity.

In conclusion, DNAm age is strongly correlated with chronological age, and appreciably more so than with telomere length and mtCN. Results from this study indicate that, even in the context of a small sample size, DNA methylation aging markers may have high precision and strong potential to detect meaningful associations with a variety of health, lifestyle, psychosocial, and neurobehavioral factors.

## Supplementary information

Supplementary Table 1. Gene identification and functional analysis using nominally significant top CpGs

Supplementary Figure1 - Boxplots GTPase activity

Supplementary Figure2 - Boxplots Cell Cell Adhesion
