# Supplementary material for: Pilot study of DNA methylation, molecular aging markers and measures of health and well-being in aging
Source: Transl Psychiatry. 2019 Mar 18;9:118. doi: 10.1038/s41398-019-0446-1 (PMC6423054; doi:10.1038/s41398-019-0446-1)
Supplement: Supplementary file 1 — Supplementary Table 1. Gene identification and functional analysis using nominally significant top CpGs [file 41398_2019_446_MOESM1_ESM.docx]

Supplementary Table:

Table 1. Gene identification and functional analysis using nominally significant top CpGs.

| **GO ID** | **GO Term (detailed descriptor)** | **GO Sub-ontology** | **Genes** | **p-value** | **Benjamini-Hochberg** |
| --- | --- | --- | --- | --- | --- |
| **GO:0090630** | **Activation of GTPase activity** | Biological Process | *NTF3, SGSM2, TBC1D9B* | 0.019788 | 0.994122 |
|  | **Descriptor:** process that initiates the activity of an inactive GTPase through the replacement of GDP by GTP | | | | |
| **GO:0098609** | **Cell-cell adhesion** | Biological Process | *MACF1, PFKP, ARHGAP18, SH3GL1* | 0.037929 | 0.993048 |
|  | **Descriptor:** attachment of one cell to another cell via adhesion molecules | | | | |
| **GO:0045664** | **Regulation of neuron differentiation** | Biological Process | *NTF3, CDK5RAP1* | 0.053415 | 0.990928 |
|  | **Descriptor**: process that modulates the frequency, rate or extent of neuron differentiation | | | | |
| **GO:0035774** | **Positive regulation of insulin secretion involved in cellular response to glucose stimulus** | Biological Process | *ANO1, FAM132A* | 0.076531 | 0.993997 |
|  | **Descriptor:** increases frequency, rate or extent of the regulated release of insulin that contributes to the response of a cell to glucose | | | | |
