# Supplementary figures and images for: Pilot study of DNA methylation, molecular aging markers and measures of health and well-being in aging
Source: Transl Psychiatry. 2019 Mar 18;9:118. doi: 10.1038/s41398-019-0446-1 (PMC6423054; doi:10.1038/s41398-019-0446-1)

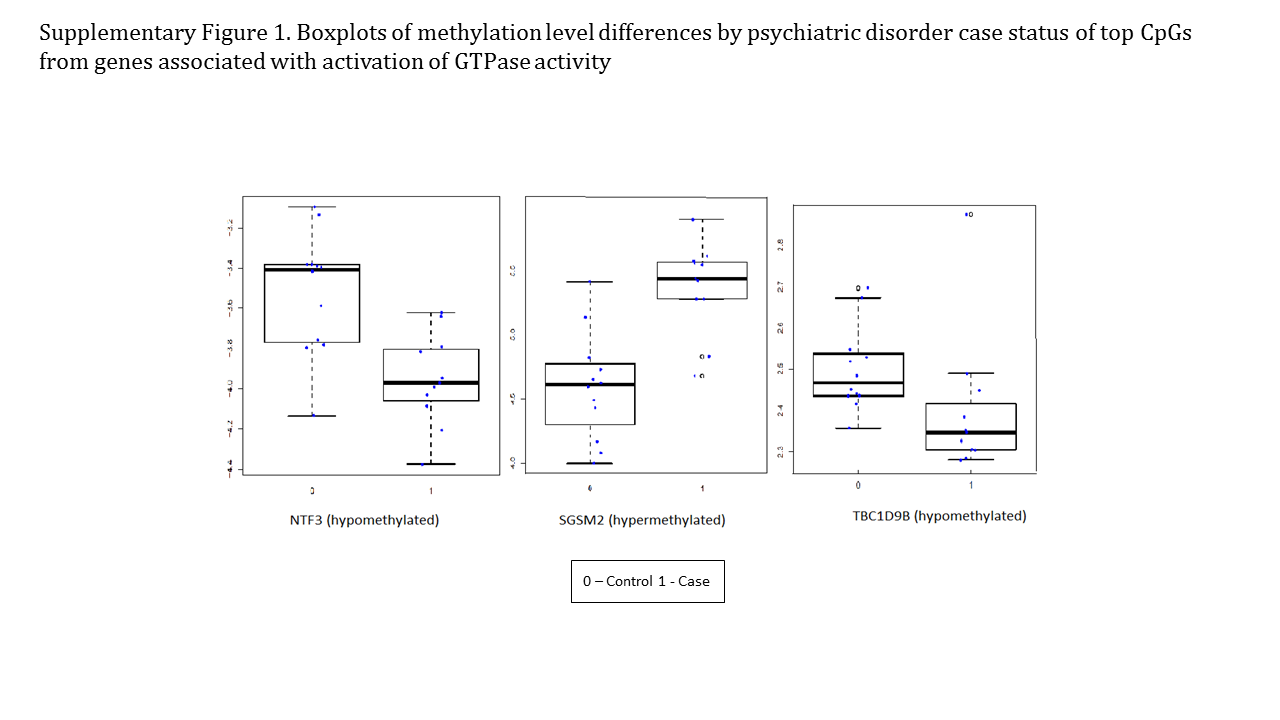

Supplement: Supplementary file 2 — Supplementary Figure1 - Boxplots GTPase activity [file 41398_2019_446_MOESM2_ESM.tif]

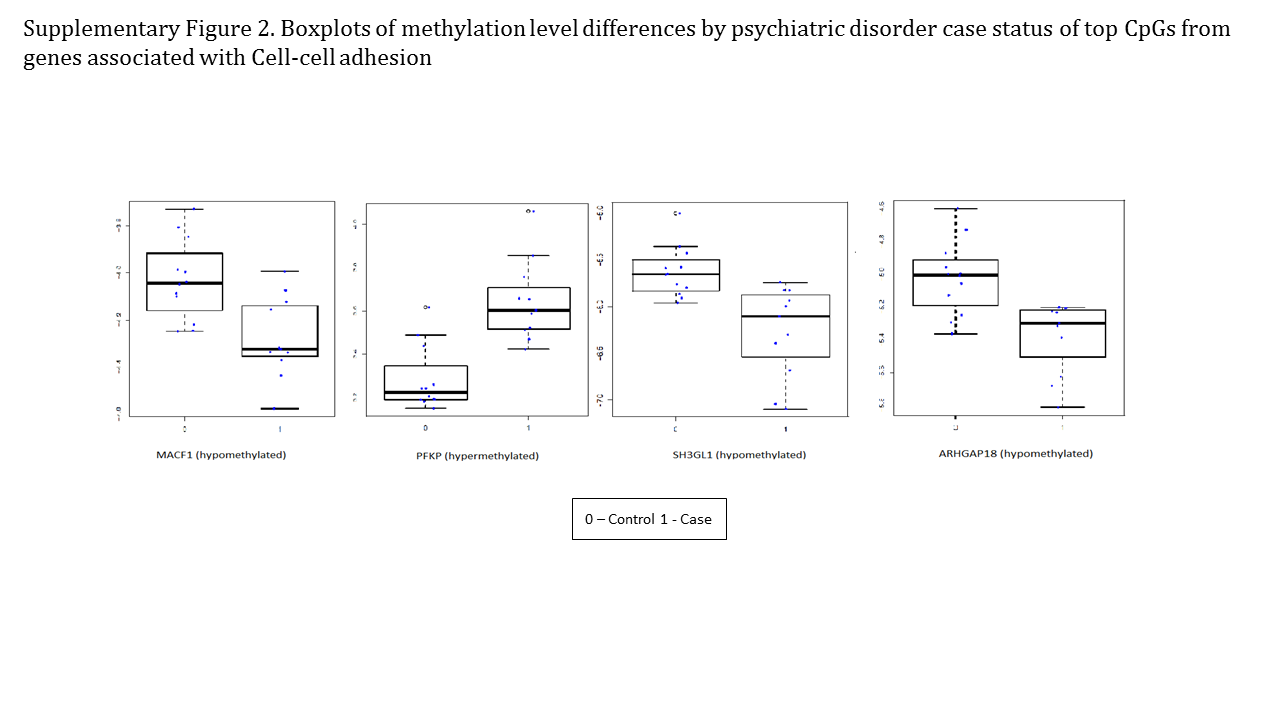

Supplement: Supplementary file 3 — Supplementary Figure2 - Boxplots Cell Cell Adhesion [file 41398_2019_446_MOESM3_ESM.tif]
